# Targeting adaptability to improve Medication Therapy Management (MTM) implementation in community pharmacy

**DOI:** 10.1186/s13012-019-0946-7

**Published:** 2019-11-27

**Authors:** Kenneth C. Hohmeier, James S. Wheeler, Kea Turner, Jarrod S. Vick, Merrill L. Marchetti, Jeremy Crain, Andrea Brookhart

**Affiliations:** 1 Department of Clinical Pharmacy and Translational Science, College of Pharmacy, University of Tennessee Health Science Center, 310 S Perimeter Park Drive, Suite 220, Nashville, TN 37211 USA; 2 Department of Health Services Research, Management & Policy, College of Public Health & Health Professions, University of Florida, Gainesville, USA; 3 College of Pharmacy, University of Tennessee Health Science Center, Nashville, USA; 4 Kroger Nashville Division, Nashville, TN USA; 5 Kroger Pharmacy General Office, Cincinnati, OH USA

**Keywords:** Adaptability, Implementation science, CFIR, Medication therapy management, MTM, CMM, CMR, TMR, Community Pharmacy, Community Pharmacist

## Abstract

**Objectives:**

(1) To develop an adaptation framework for MTM delivery for pharmacists (the MTM Adaptability Framework), (2) to examine the impact of an educational intervention informed by the MTM Adaptability Framework on MTM completion rates over a 2-year period, and (3) to explore pharmacists’ perceptions regarding knowledge and beliefs about MTM and MTM implementation self-efficacy pre- and post-intervention.

**Methods:**

This study is a prospective, mixed-methods research study including a quasi-experimental, one-group pretest-posttest quantitative study with a sequential explanatory qualitative study arm featuring semi-structured key informant interviews. US supermarket pharmacy chain setting included 93 community pharmacy sites located in Tennessee, Kentucky, and Alabama. MTM completion rates are reported as percentage of completed comprehensive medication reviews (CMRs) and targeted medication reviews (TMRs) and pharmacist perceptions.

**Results:**

An 11.4% absolute increase in MTM completion rates was seen after the educational intervention targeting adaptation of MTM in the community pharmacy setting. This was found to be significant (46.92% vs. 58.3%; *p* < 0.001). Responses to the semi-structured interviews were mapped against CFIR and included themes: “knowledge and beliefs about MTM (pre-intervention),” “self-efficacy for MTM implementation (pre-intervention),” “knowledge and beliefs about MTM (post-intervention),” and “self-efficacy for MTM implementation (post-intervention).” Data convergence was found across these methodologies and suggested that targeting adaptability of MTM delivery increases MTM completion rates (quantitative data) and positively changes perceptions of MTM feasibility and self-efficacy (interviews).

**Conclusion:**

The use of an educational intervention about adaptation of MTM to influence adaptation of MTM to a chain community pharmacy setting part of an implementation strategy improved MTM completion rates significantly. Future research should investigate combined implementation strategies and their impact on MTM implementation success.

Contributions to the literature

- Practitioners often receive guidance on how to implement evidence-based interventions but lack guidance on how to adapt evidence-based interventions. Providing guidance on how to adapt evidence-based interventions can help practitioners to anticipate or plan for adaptation prior to implementation.
- To date, few studies have tested educational interventions that support adaptation and whether such interventions positively affect implementation outcomes, such as program reach (penetration) and providers’ implementation self-efficacy.
- Providers’ may have unrealistic expectations about the time and resources needed for implementation of evidence-based interventions, such as medication therapy management, decreasing their self-efficacy for implementation. Therefore, providing an educational intervention on adaptability may improve providers’ knowledge and beliefs regarding implementation self-efficacy.
- When provided with an educational intervention supporting adaptation, an increase in medication therapy management implementation success was seen, providing initial evidence that an educational intervention focused on adaptability can improve implementation success.
- The findings from this study address key gaps in the literature, including the development of an adaptation framework for pharmacy settings and medication therapy management interventions and testing the impact of an education intervention that supports adaptation on implementation outcomes, such as program reach.

## Background

Medication therapy management (MTM) involves a range of services to optimize patients’ medication regimen and at the same time, detect and prevent potentially costly medication errors [1]. MTM services can be offered by pharmacists or other qualified providers and are incentivized through programs, such as the Medicare Part D MTM program. Pharmacists are particularly effective at leading MTM services given their doctorate-level training and scope of practice. The Centers for Disease Control and Prevention considers pharmacist-led MTM services to have strong evidence of effectiveness [2]. In the U.S., MTM has been tested in a variety of contexts and patient populations and demonstrated positive effects on medication adherence, clinical outcomes, and safety (e.g., reduction in therapeutic duplication) [3–9]. Over the past decade, MTM adoption in the US has grown; by 2015, 81% of independently owned pharmacies reported offering of MTM services [10]. Internationally, MTM is also growing. For example, a recent survey in Europe found that 44% (11 of 25 countries surveyed) provide adherence-focused MTM services, while 24% (6 of 25 countries surveyed) provide comprehensive MTM services similar to those in the USA [11].

MTM service expansion now receives bipartisan support from US lawmakers and their constituents [10–13]. Despite increased adoption and support, studies suggest that MTM implementation is inconsistent across settings [14]. For example, a recent Centers for Medicare and Medicaid Services (CMS) report highlighted low completion rates (annual number of paid claims versus total number of patients eligible for the service) for Comprehensive Medication Review (CMR) opportunities at 15.4% for Prescription Drug Plans and 30.9% for Medicare Advantage Plans [15].

Researchers have suggested that inconsistent MTM implementation stems from inconsistent definitions of MTM, lack of clarity regarding the core components of MTM, and lack of implementation support to assist pharmacists with adapting MTM [13]. Last addressed in 2004, the broad consensus definition for MTM developed by seven major national pharmacy associations is “a service or group of services that optimize therapeutic outcomes for individual patients” [16]. However, since that time, payors, legislators, academics, professional organizations, and practitioners have added complexity to the concept of MTM. Such ambiguity surrounding MTM terminology [17–19] and continued debate surrounding what constitutes MTM [18–20] may be directly and negatively impacting MTM implementation and setting-specific adaptation. In an attempt to alleviate this issue, the pharmacy profession established Core Elements for MTM services: medication therapy review (MTR), personalized medication record (PMR), medication action plan (MAP), intervention and/or referral, and documentation and follow-up [12].

Importantly, the Core Elements framework “does not represent a specific minimum or maximum level of all services that could be delivered by pharmacists” [21]. Furthermore, although the use of the five elements is required, the delivery of these elements may vary by setting [12]. For instance, a pharmacist must review the patient’s medication regimen, but if a medication-related problem is identified, the Core Elements do not specify when a pharmacist should intervene versus refer or what factors a pharmacist should consider in making that decision. One specific example of this phenomenon can be understood when considering the same pharmacist providing MTM to a patient with uncontrolled hypertension in (a) a community pharmacy as compared to (b) a patient-centered medical home (PCMH), including access to the entire patient’s medical record and care team. In both instances, the pharmacist identifying the problem is responsible for its resolution and has the same level of clinical expertise, however given the setting of the community pharmacy an intervention to directly alter the medication regimen is less likely to be successful given the lack of full patient record access and inability to communicate easily with the care team—consequently, a referral would be a more appropriate intervention. To assist with MTM implementation, pharmacists need guidance on how to adapt MTM for their specific setting. There are frameworks available for adapting interventions; however, there are currently no frameworks available for pharmacy settings [22–27].

Rogers first described adaptation or reinvention as the extent to which attributes of an innovation are changed during the adoption and innovation process [28]. Rogers suggested that adaptation can be unplanned (e.g., adopter’s lack of knowledge about the innovation) or reactive (e.g., changes made due to problems encountered during implementation) [28]. Since that time, many researchers have distinguished planned versus reactive adaptations and adaptations that enhance or decrease program effectiveness (e.g., program drift) [29–33]. More recently, researchers have developed coding systems for adaptations—to distinguish adaptations by who is making the modification, what intervention attribute is being modified (e.g., content versus context), and what level of the delivery system is being targeted (e.g., individual- versus system-level). Adaptation can also be characterized as a process that may change over different phases of implementation [22] and is impacted by multi-level factors within and outside of the organization’s context [34]. Given how complex intervention adaptation is, researchers have called for the development of adaptation frameworks to assist practitioners with adapting evidence-based interventions to preserve the core components of the intervention and retain program effectiveness [35]. While several adaptation frameworks have been developed—specifically, in HIV, substance use, and child welfare settings—guidance is needed for pharmacists implementing MTM, which is a complex intervention with several core components and has historically been defined inconsistently across settings.

Studies have documented notable barriers to MTM implementation but have not discussed the issue of adaptation. For instance, much of the literature on barriers to MTM implementation cites reimbursement, staff support, and time [36, 37]. However, to date, there has been no published research regarding implementation strategies for supporting adaptation. This ability to determine how MTM fits into an existing workflow with known constraints like communication with prescribers and access to patient medical information represents a novel target for an implementation strategy to impact MTM completion rates. Poor adaptation of MTM based on setting-specific factors may in part explain why staff support and time are frequently listed in the literature as barriers to MTM implementation. Studies have shown the value of providing implementation support to pharmacists implementing MTM. For example, a recent study found that implementation support (via coaching and increased centralized-support) compared to financial incentivizes was more effective in supporting pharmacists with MTM implementation [38]. Therefore, it is possible that implementation support along with guidance about adaptation may assist pharmacists with MTM implementation.

## Objective

To address this gap in the research, our study has three aims: (1) to develop an adaptation framework for MTM delivery for pharmacists (the MTM Adaptability Framework), (2) to examine the impact of an educational intervention informed by the MTM Adaptability Framework on MTM completion rates over a 2-year period, and (3) to explore pharmacists’ perceptions regarding knowledge and beliefs about MTM and MTM implementation self-efficacy pre- and post-intervention.

## Methods

This study is a prospective, mixed-methods research study including a quasi-experimental, one-group pretest-posttest quantitative study with a sequential explanatory qualitative study arm featuring semi-structured key informant interviews. To enhance finding validity, in addition to separate analysis for each study arm, a parallel mixed-methods analysis was performed to identify data convergence or divergence and reported in the results section [24]. The study was approved by the University of Tennessee Health Science Center’s, Institutional Review Board (IRB).

### MTM adaptability framework theoretical underpinnings

To simplify the pharmacist’s decision-making process when approaching an MTM session, the authors set out to develop and validate a framework for adapting MTM services. The MTM Adaptability Framework was founded upon previously published MTM frameworks and incorporates recent additions to MTM terminology and changes to pharmacy practice [2, 22, 34–38]. In particular, it does not create a new MTM service—rather it makes use of existing and accepted terminology and consensus definitions to simplify and connect concepts related to MTM. The framework aims to do as follows:

1. Compare the scopes of various direct patient care services provided by pharmacists
2. Align pharmacist-provided direct patient care services (i.e. MTM) on a spectrum from low to high intensity of service delivery
3. Provide a structure to systematically approach the delivery of each type of MTM service

Initial development of the framework was based on experiences of organization leadership, front line pharmacy staff, and university researchers. An overarching area of concern of the expert panel was the pharmacist’s approach to delivering MTM services varied widely across pharmacies within the organization and with an “all or nothing” mindset. Specifically, it was noted that levels of effort in delivering MTM services did not align with the MTM service being requested, pharmacists had low self-efficacy in providing services despite baseline training, and pharmacists lacked a firm grasp on MTM delivery logistics. These findings are further supported by recent studies in similar settings linking the confusion surrounding implementation of new patient care services in the community pharmacy to reduced self-efficacy and lower implementation rates [38–40].

The barriers to MTM provision identified by the expert team were mapped to the CFIR to aid framework development and validation strategy. The overarching construct of “pharmacy staff characteristics” was selected, along with related sub-constructs: (a) knowledge and beliefs about the intervention and (b) perceived self-efficacy [34, 39]. Knowledge and beliefs about the intervention are primarily a cognitive function that bridges knowledge about the intervention with the ability to apply and adapt the intervention (MTM) to their practice site [28]. Providers must have general knowledge about the intervention (e.g., MTM requirements), procedural knowledge (i.e., how to carry out the steps of MTM), and knowledge of the task environment (i.e., how the outer context, such as patient preferences and needs, might affect implementation) [41]. Without effective training, rejection and discontinuation of the intervention are likely [42]. Similarly, a provider’s individual belief in their own capabilities to perform the intervention (e.g., implementation self-efficacy) is central to implementation success and commitment to the intervention, especially in the face of obstacles [43]. Therefore, a training program was chosen to disseminate, inform, and validate the MTM Adaptability Framework [43].

In addition to targeting knowledge and beliefs, the theory of planned behavior (TPB) was subsequently used to form both the framework and training program design, including identifying specific learning objective targets (attitude, subjective norm, and perceived behavioral control) [44, 45]. Attitudes, or ascribing a value to a particular outcome (e.g., likelihood of improving patient outcomes through MTM), can affect a provider’s likelihood of implementing MTM [43]. Given how many services can be implemented with a community pharmacy (e.g., vaccination, smoking cessation, MTM), pharmacists’ assessment of the value of a given service is likely to affect their willingness to prioritize and implement that service. Additionally, whether a provider believes a behavior, such as MTM implementation and adaptation, is within their control, is likely to influence their implementation of MTM. MTM is a complex intervention with multiple core components and it is likely that perceived behavior control is a critical determinant in MTM implementation. Social pressure—such as subjective norms—is also an important determinant of behavior change, particularly in community pharmacies. Community pharmacies are often under significant financial pressure for complex reasons (e.g., declining direct and indirect remuneration) and strive to be market competitive by offering clinical services that have been implemented in other high-performing community pharmacies.

### Project setting

The intervention took place in a division of a US supermarket pharmacy chain setting, which included 93 community pharmacy sites located in Tennessee, Kentucky, and Alabama. The chain pharmacy has been involved in MTM for over a decade. As a part of their job duties, staff pharmacists were expected to perform MTM services within workflow and MTM practice had been promoted as a strategic goal for the company.

### Intervention and strategies

To address suboptimal implementation rates within the study pharmacy organization, an educational intervention was developed based on experiences of organization leadership, front line pharmacy staff, and university researchers. The training intervention was a 60-min live training webinar with active learning components that was jointly developed by university researchers and organizational leadership. The overarching goal of the training was to provide guidance to community pharmacists on adapting the delivery of the MTM Core Elements to a Community Pharmacy setting.

The training included both didactic and case-based delivery of the following principles: (1) MTM time management training, (2) definitions of the scope and purpose of each MTM service based on national professional and payor definitions (e.g., implementation glossary), and (3) the use triage and referral based on the following:

1. The type of MTM service being provided
2. Pharmacist access to medical information
3. The pharmacist’s role within the patient’s healthcare team

Core to the training program was the concept of “feasibility” of MTM within community pharmacy workflow. To establish a new “subjective norm,” the webinar used examples of high-performing pharmacists within the same organization integrating MTM feasibly into their workflow. Other approaches used in the webinar included both knowledge shaping (instruction on how to perform behavior and re-attribution of perceived barriers) and fostering self-belief (verbal persuasion of capability and information about antecedents).

Finally, a framework was introduced to pharmacists presenting standardized guidance to customize their MTM delivery approach to the various types of MTM services provided at their pharmacy. The framework was first presented at a high level and was followed by specific examples, which applied the theoretical framework. For example, comparisons were made between the level of service required of a CMR (e.g., MAP, PMR, and MTR) versus what a pharmacist may perceive as the level of service (e.g., therapy changes under CPA, laboratory value assessment, disease state education, questionnaire distribution) and the feasibility of each (also see Additional file 1: Supplement S1).

At least one pharmacist from each of the 93 pharmacies in the local supermarket pharmacy chain division was required to attend the educational webinar. Four live sessions were broadcast over a 2-day period. As an incentive for participation, pharmacists earned 1 h of educational credit for activity completion.

### MTM program reach

The primary outcome for the study was MTM program reach which was reported as a proprietary internal metric used by the supermarket pharmacy chain known as the Net Effective Rate (NER). The NER is a type of MTM completion rate and is defined as the number of TMRs and CMRs successfully completed divided by the total number of opportunities that are available across three national MTM vendors (OutcomesMTM, Mirixa, and SOCRxATES). Successful completions were defined as provided CMRs and TMRs adjudicated for payment with the MTM vendor.

### Semi-structured interviews

Key informants were recruited from the list of trained pharmacists provided by chain pharmacy leadership to provide broader context and findings not readily obtained via MTM completion rate data. Interviews were continued until a point of saturation was achieved, a point at which researchers concluded no further new information was obtained. A total of 8 participants were interviewed for this study between May and June 2017. No honorarium or other incentive was provided. An initial draft of the interview questions was developed internally by the research team in collaboration with pharmacy leadership using responses from open-ended survey questions. The final interview guide included 2 domains of inquiry, perceptions before and after the educational intervention, and 5 questions (Additional file 1: Table S2). The interview guide was intentionally kept brief to ensure short interviews that could be conducted during workflow. KH (experienced in qualitative research) and JV conducted the recorded semi-structured interviews with the participants via telephone and face-to-face depending on respondent availability or preference. At saturation, 8 pharmacists had been interviewed (5 men and 3 women), with representation from several districts across regional organization division. Interviews ranged from 12–31 minutes.

### Analysis

For the analysis of the quantitative arm of the study, the a priori level of significance was set a priori at *p* < 0.05. For the primary outcome, an a priori power analysis indicated 64 pharmacy sites would be required for 80% power to detect a medium-sized effect with a level of significance defined at 0.05. Pre- and post-intervention MTM completion rates were compared between the 4-month periods of December 2015 to April 2016 and December 1, 2016, to April 30, 2017, via paired *t* test.

The interviews were recorded, transcribed, and entered into a qualitative analysis software program (NVivo qualitative data analysis Software; QSR International Pty Ltd. Version 10, 2014). Data were analyzed using a constant comparison technique whereby analytical coding procedures begin before the data collection has been completed rather than in a post hoc manner. Two researchers (the principal and one co-investigator) independently coded the data from the first five interviews deductively using CFIR as a framework [5, 14]. The researchers then came together to compare their independently generated themes, discuss potential emerging themes, and reach agreement on a coding structure before further analysis. They then coded the remaining interviews independently and then again worked collaboratively to refine and organize the categories of themes in the data.

## Results

Demographics of enrolled pharmacy study sites and key informants are found in Table 1. In total, 93 pharmacies across 3 states (Tennessee, Kentucky, and Alabama) were included in the study. Interviews were conducted with 8 pharmacists who had undergone the educational intervention training.

**Table 1:** Demographics for study sites and key informants

	Number	Percentage (%)
State		
Tennessee	81	87.1
Alabama	8	8.6
Kentucky	4	4.3
Sex		
Male	3	37.5
Female	5	62.5
Degree		
PharmD	4	50
BPharm	4	50

*PharmD* Doctor of Pharmacy, *BPharm* Bachelor of Pharmacy

### MTM adaptability framework

**Fig. 1:**
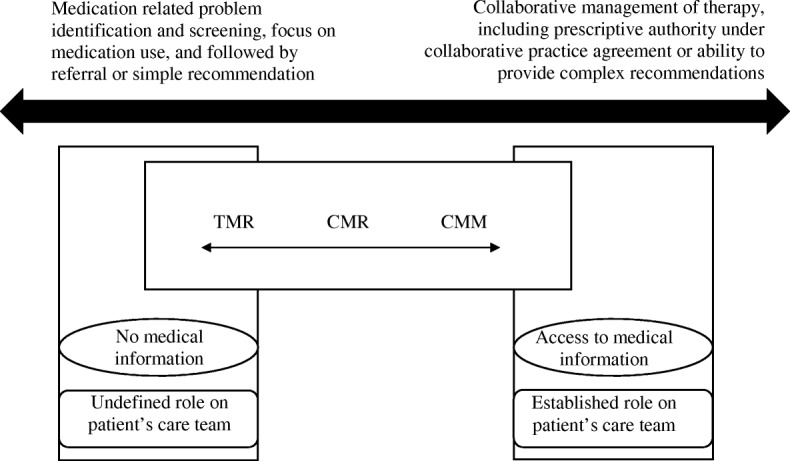
Spectrum of MTM services

**Fig. 2:**
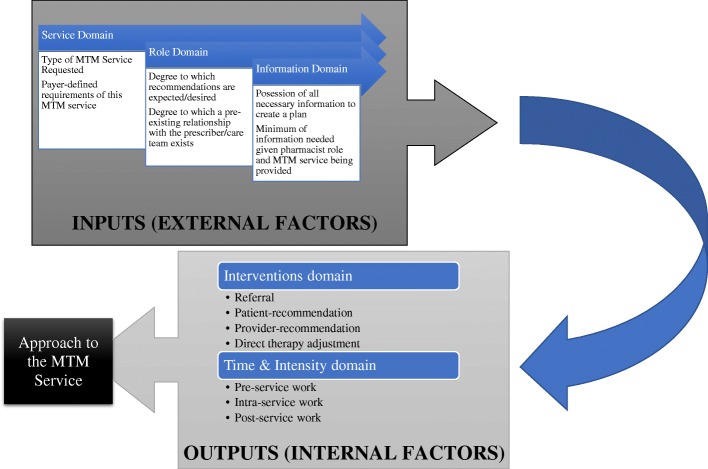
MTM Adaptability Framework

The aim of the MTM Adaptability Framework was to create a framework for adapting MTM services. The framework presented here compares the scopes of various direct patient care services (i.e., MTM) provided by pharmacists, aligns these MTM services on a spectrum from low to high intensity of service delivery (Fig. 1), and provides a structure to systematically approach the delivery of each type of MTM service (Fig. 2). To best incorporate the concepts discussed above into a single, unifying framework and given the results of the present study, the MTM Adaptability Framework is composed of three inputs and two outputs (Fig. 2).

The three domains classified as “inputs” form the pharmacist’s approach to MTM: service domain, role domain, and information domain. These domains are external to the pharmacist and beyond their control or influence and may be thought of as independent variables. Within the service domain is the explicit definition and goals of the service being provided. As the largest MTM payor in the USA, CMS has provided definitive language within Medicare Part D rules on MTM service definition and level of effort [46, 47]. MAPDs and PDPs must provide two distinct MTM services: an annual CMR and periodic targeted medication review (TMR). Medicare sets the expected level of care unambiguously in its definitions of each of these two services. First, the CMR is defined as “review of the individual’s medications, which may result in the creation of a recommended medication action plan with a written or printed summary of the results of the review provided to the targeted individual” with the aim to “aid in assessing medication therapy and optimizing patient outcomes” [48, 49]. The second service, the TMR, is different from the CMR in that “while the follow-up intervention that results from a TMR may be person-to-person, the TMR is distinct from a CMR because it is focused on specific actual or potential medication-related problems, and a CMR is a comprehensive, real-time, interactive medication review and consultation with the beneficiary to assess their medication use for the presence of medication-related problems and results in the creation of a written summary in CMS’ standardized format.”

Given that neither the TMR nor CMR captures the pharmacist’s full complement of skills in improving patient outcomes, the American College of Clinical Pharmacy (ACCP) established the concept of comprehensive medication management (CMM) [22]. This distinct service includes more frequent follow-up, accountability for achieving clinical outcomes, and is typically provided in direct collaboration with the patient’s health care team. In addition to this service domain, a pharmacist must concurrently consider both their role (established or not) on the patient’s health care team and what medical information is available to them about the patient (laboratory values, histories, etc.).

Given these inputs, a pharmacist should next plan for both the time and intensity spent on the specific MTM service, as well as the need for referral to other services offered by the pharmacy, or other providers. These domains are referred to as “outputs,” and may be thought of as items within the pharmacist’s control (dependent variables). Depending on the varying goals across the MTM service spectrum (CMR, TMR, CMM), the role on the patient’s health care team, and information available at the time of the service, a pharmacist’s interventions may be as simple as a referral to the patient’s primary care provider—or as complex as a therapeutic addition, modification, or discontinuation via collaborative practice agreement (CPA). The understanding of which services are the least time and resource intensive (e.g., TMR), moderately time and resource intensive (e.g., CMR), and highly time and resource intensive (e.g., CMM) provides a foundation from which the pharmacist is able to use resources efficiently and effectively in line with the goals of the Core Elements [2]. And, understanding the goals of the service being provided, what information is available from which to base interventions from, and the pharmacist’s role on the care team provides the necessary inputs for a pharmacist to make realistic and feasible interventions that reduce patient, provider, and pharmacist confusion and burden whilst within workflow (see Additional file 1: Supplement 1).

### MTM completion rates

Power was met with a total of 93 sites enrolled in the study. A statistically significant 11.4% absolute increase in MTM completion rates was seen after the educational intervention, when comparing 20 weeks of data post-intervention to the equivalent time period the prior year (46.9% vs. 58.3%; *p* < 0.001).

### Semi-structured interviews

Themes included “knowledge and beliefs about MTM (pre-intervention),” “self-efficacy for MTM implementation (pre-intervention),” “knowledge and beliefs about MTM (post-intervention),” and “self-efficacy for MTM implementation (post-intervention).” In addition, a theme named “divergent perceptions” and included opinions of those key informants who perceived no benefit from the intervention.

#### Theme 1: Knowledge and beliefs about MTM (pre-intervention)

This theme was specific to initial MTM adaptation intentions and beliefs prior to training. Key informants noted that initially, they had perceived a lack of time to integrate MTM into their practice. Lack of staff was also noted as a major barrier to implementation.

I felt like I had to fix every problem I came across especially when doing a CMR… (P2)

“I would to say again the time was the largest barrier, thinking that I had to devote 30 minutes to an hour to each one to do it and how much time that took me away from all my other responsibilities.” (P2)

#### Theme 2: Self-efficacy for MTM implementation (pre-intervention)

Pharmacists noted how the educational intervention induced paradigm shifts in how they perceived their own ability to conduct an MTM session within pharmacy workflow—so-called “self-efficacy.” This was associated with the educational intervention objectives of time management training, defining the scope and purpose of each MTM service, and the use of triage and referral. Pharmacists overall reported that prior to the intervention they were bought-in and supportive of MTM and viewed it as having a positive impact on patient care; however, they concurrently felt that they themselves could not feasibly integrate MTM at their practice site. Informants also noted that they perceived MTM, especially the CMR, as a complex and time-intensive service not suitable for a community pharmacy setting, and this perception changed after the educational intervention.

Actually, I thought [MTM] was a great idea. It’s what we’re trying to do. My reservation was that we were already very busy doing other activities and to add this to our normal routine was difficult. I don’t feel like I have enough time to thoroughly go over all the information that I need for the patient and do my regular job. (P6)

[MTM] was a little bit too time consuming and that it was hard to fit in my current schedule. (P2)

#### Theme 3: Knowledge and beliefs about MTM (post-intervention)

Pharmacists noted spacing out problem resolution over several follow-up sessions or referring patients to other clinical services offered by the pharmacy to fix a problem (such as referring a CMR patient to a diabetes disease state management program) as helpful. Furthermore, pharmacists described paradigm shifts around the idea of triage and referral—whereby identified patient problems should be triaged prior to developing and implementing a plan, with some problems being referred to other members of the health care team for follow-up (such as the prescribing physician). Pharmacists also compared and contrasted the time they spent on different types of MTM services before and after the training, highlighting where they fit on the “Spectrum of MTM Services” (Fig. 2). For example, pharmacists would describe most CMR sessions ranging from 20–30 min after the educational intervention (from upwards of 60-min sessions prior to the educational intervention)—although this was said to vary based on the patient and may exceed those limits in some circumstances. In particular, pharmacists noted they were often providing CMM-level care intensity and time, when CMR-level services were requested. In other circumstances, pharmacists noted that the use of face-to-face time with the patient should be for collecting information and assessing the patient, but that sometimes based on workflow demands, planning and implementing may have to occur after the appointment and when time allows based on fluctuating workload demands. Lastly, pharmacists alluded to the need for explicit directions and directions on how new MTM services should fit into the workflow of their organization—including time management and service scope and goals when delivering the service.

I think my interpretation of an MTM was you had to go over every possible issue that affects the patient [during the first visit]… I think seeing the example where the person [was] conducting an MTM was really helpful; to see how she sort of narrowed it down and just focused on certain issues. (P3)

I think that’s where it’s confusing, where the terminology comes into play with TMR’s or so CMR’s versus CMM. So, I think, pharmacists go into it expecting every single time to be doing a CMM (P4)

… we’ll discuss a [medication therapy problem of] ‘needs therapy’ and things like take that we’ll ask them to follow up with their physician. Most of those people, had an appointment coming up in a next couple of months anyway. And oftentimes, we provide them with the remainder sheet to take with them, too to discuss it [with their prescriber at the next visit]. (P2)

#### Theme 4: Self-efficacy for MTM implementation (post-intervention)

Those interviewed indicated this paradigm shift in self-efficacy was mostly attributed to gained knowledge of the spectrum of MTM services and in particular, the different intensity and goals (TMR, CMR, and CMM). After the educational intervention, the pharmacists noted that adaptations made to their MTM approach improved self-confidence and perceived ability to deliver services at their pharmacy. Such altered approaches included limiting the CMR session to CMR-specific session priorities (medication-related priorities such as identifying medication-related problems), time management (less time on diet, lifestyle, and disease management), and referral for those items not readily addressed in the community pharmacy (physician referrals for further workup or therapeutic adjustments). Subsequently, pharmacist informants noted a changed perception of their own ability to integrate MTM into their pharmacy workflow. It should be noted that even with improvements to self-efficacy and subsequent MTM implementation, pharmacists still noted feelings of being both short staffed and rushed when integrating MTM into their pharmacy’s workflow.

So, everyone kind of knows that it’s a great thing and it’s a great opportunity but for some people it might feel unattainable because I feel like they’re trying to put that too much into it. (P4)

[The educational intervention] really created a lot of things in my head. I was like, okay this is not as overwhelming as it seems to be. It really opened my eyes, I guess to the fact that we didn’t have to do everything in a TMR or CMR ... When I brought that back to my staff, it really made all of us feel a lot more at ease and we had a lot of success at completing these things then, especially targeting patients who have prescriptions ready… (P2)

I think the perception has improved significantly. They don’t feel like they have to take 30 minutes or an hour of their day. (P2)

Lastly, a theme including “divergent perceptions” included minor themes reported by pharmacists which differ from major themes previously described. These minor themes are reported to provide a comprehensive view of pharmacist thoughts, feelings, and perceptions related to MTM implementation. Staffing-related issues presented a persistent barrier to some even after the educational intervention and adaptations made to their approach to delivering MTM. In other circumstances, pharmacists noted that no improvements to MTM delivery were possible as a lack of staff help made it impossible to provide any level of MTM. Staff help included both technicians, as well as pharmacist overlap (when more than one pharmacist are on duty at one time). Some pharmacists also noted that training was valuable and improved overall MTM completion rates, but that greater gains might be made if there were concurrent efforts to shift in the team’s perceptions of MTM delivery and its relative importance.

I still feel the same way [about feasibility as before the intervention]. It’s just really difficult when you-- if I had some extra help and in fact days where extra help is sent to our store, an extra pharmacists, it does give me a chance to address other activities that we’re to be doing… there’s just many other things beside filling prescriptions that are happening in the pharmacy so trying to work all of that in with one pharmacists and multiple patients is not easy. (P6)

We have increased our MTM percentage here at this particular store, [it is still] not very good, it’s poor. And it has increased since the webinar but like I said it’s still just the main-- just getting everybody on board and trying to get a routine established [that is the problem]. (P8)

### Data integration results

Two separate methodologies were used to investigate the impact of the education intervention: quantitative pre-post quasi-experimental and qualitative semi-structured interviews. Data convergence was found across these methodologies and suggested that targeting adaptability of MTM delivery increases MTM completion rates (quantitative data) and positively changes perceptions of MTM feasibility and self-efficacy (interviews). Although this impact was found across the majority of individuals, the intervention’s impact was not absolute across all trained pharmacists. This idea of an intervention refractory subgroup is supported by minor themes identified in the qualitative, semi-structured interview arm of the study.

## Discussion

The purpose of this study was to develop and test an adaptation framework for MTM. This the first study to our knowledge to develop an adaptation framework for a pharmacy setting. Additionally, few studies have explored how adaptation affects practitioners’ knowledge, beliefs, and self-efficacy for implementation [48]. The results demonstrated that indeed such an intervention was able to produce statistically and clinically significant improvements in MTM completion rates (TMR and CMR) after training. These improvements were consistent with absolute effect sizes of 6–13% seen with other implementation strategies within established health care practice settings [49] Therefore, these results support the addition of implementation strategies involving adaptability as part of a pharmacy organization’s MTM implementation plan.

Despite overall improvements in MTM completion rates and changes in pharmacist perceptions, MTM implementation improvements were not seen equally across all sites. This gap is further evidenced by the published literature [33, 35]. Both the moderate degree of MTM completion rate improvement and presence of a refractory subgroup further highlights the fact that there is no “magic bullet” to improving the implementation of MTM, and that a holistic approach to developing an implementation strategy within an organization should include multiple approaches. This intervention sought to promote adaptability—by guiding pharmacists on how to adapt their MTM delivery approach to align with the existing services within the pharmacy [50, 51]. Additionally, this intervention developed an implementation glossary—to ensure that participating pharmacies had a shared understanding of what MTM implementation means. Future studies could test whether additional implementation strategies, such as providing ongoing consultation to address questions that may arise after the initial training, strengthen the effects of this educational intervention on MTM implementation outcomes, such as reach and fidelity. Additionally, barriers outside of a pharmacy organization’s control still remain significant obstacles to widespread MTM implementation, such as widespread reimbursement for pharmacist-provided services, CMS policies and incentives, prescriber and patient perceptions, and legal authority and scopes of practice [34, 36, 37, 39]. Despite these barriers, pharmacy leaders and managers should not overlook those implementation factors within their control, such as assisting in the adaptation of new clinical services, self-efficacy, and perceived feasibility when implementing their MTM services.

The current study also suggests that MTM implementation is negatively impacted by ambiguity and confusion surrounding MTM terminology and delivery approach. This study highlights how some barriers to MTM implementation are within the control of the profession as a whole, and others are within the control of individual pharmacists in particular. One’s “approach” to care delivery is distinct from their clinical capabilities and technical abilities to navigate MTM platforms, document care, or work up patients. Given the relative newness of direct patient care services in the community pharmacy and relative lack of formal modeling or instruction in how one approaches MTM service delivery, it is no wonder why “approach confusion” may occur. It is likely that such “approach confusion” creates the perfect storm for the overextension of pharmacist’s time and resources beyond the scope of the service being provided [15]. And, such feelings of being overwhelmed are well-known to reduce the implementation of health care services in other professions [52–54].

**Fig. 3:**
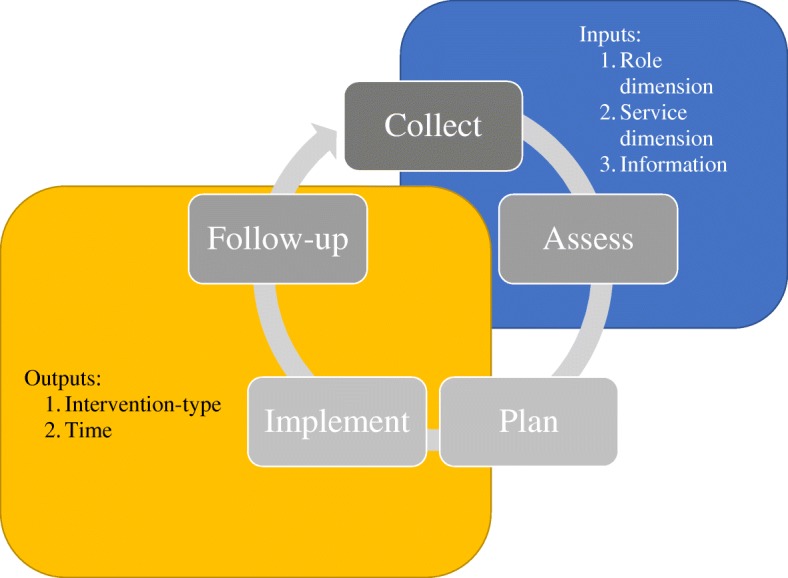
Framework mapped to the PPCP

In order to simplify “approach confusion,” the present study also introduced the MTM Adaptability Framework as a medium for educators, leaders, and managers to assist front line community pharmacists in systematically approaching the various forms of MTM at their organization. This framework is all the more important given the call for a clear, unifying message on MTM has been made for over the past decade from front line pharmacists, leaders in the profession, and academic researchers [12, 20, 21, 46, 47, 55]. It is important to mention that the Pharmacists Patient Care Process’ (PPCP) continues to serve as the overarching framework for pharmacist-provided patient care, and its importance for the profession cannot be overemphasized as pharmacists continue to pursue provider status and a greater role in the care of patients. It is the authors’ belief that the novel framework presented here should be used in conjunction with the PPCP, and not as a replacement. However, the MTM Adaptability Framework adds further depth to PPCP in terms of planning, implementing, and following-up across multiple settings where access to medical information, the role of the pharmacist, and types of services being provided vary. Given this relationship, it is possible to map the PPCP to the Consolidated Framework (Fig. 3).

Triage and referral were core components of the educational intervention and were noted in the qualitative explanatory arm of the study as important concepts for improving self-efficacy in MTM implementation. First presented in the MTM Core Elements, triage, and referral have long been supported as an important and appropriate intervention for pharmacists providing MTM services [12]. As housed in the MTM Adaptability Framework, the concept of triage and referral is all the more important to pharmacists who practice in settings without real-time access to medical records or without an established role on the patient’s health care team—as is the case for most community-based pharmacist practitioners (CPP) [56]. These CPPs have a unique skill set in providing direct patient care and practice in a variety of community-based settings. Although, in many instances, CPPs are capable of making medication changes to optimize a patient’s health due to their training, it would be impractical for them to make regimen changes without a clearer picture of the patient’s overall health or an established relationship with the patient’s provider. In these cases, a referral to a primary care provider (PCP) for further workup is as important an intervention as prescribing a medication or ordering a lab. Despite this, referral and triage are often not chosen as interventions. This may be because pharmacists allow their clinical expertise and knowledge to determine the level and intensity of the intervention and do not consider other important factors such as role on the care team, the goals of the MTM service being delivered, and the available patient care information in front of them. This “stigma” of referral as an intervention that is below the pharmacist’s skillset, or the feeling of the need to “do something” after an MTM appointment, should be dispelled as it is a well-established technique taught and practiced by colleagues across other health disciplines.

There were limitations to the study. The intervention was provided within a single national supermarket pharmacy chain division in the southeast region of the USA. Additionally, due to the real-world setting in which the intervention took place, it is possible that confounding variables may have contributed to the increased MTM completion rates. A well-known variable that is known to increase MTM completions in the community pharmacy setting is the annual fluctuation in available cases and requirement to finish these cases by the end of the fiscal year. For this reason, the study compared two equivalent time periods in two consecutive years. To ensure that all confounding variables were fully accounted for, discussions with corporate pharmacy leadership and monitoring of changes to corporate MTM metrics and bonus structures occurred during and after the study. At the completion of the study, no changes to bonus pay structure related to MTM completion rates, additional MTM training, or new penalties for missing MTM completion metrics occurred.

## Conclusion

The use of an educational intervention on MTM adaptability as part of an implementation strategy aiming to improve MTM completion rates was found to be clinically and statistically significant. Organizations interested in improving MTM service implementation should include interventions which address how a pharmacist adapts MTM delivery to their unique pharmacy setting as part of their implementation strategy. However, this implementation strategy should be used in combination with other proven implementation strategies whenever possible to maximize impact. Future research should investigate such combined implementation strategies and their impact on MTM implementation success.

## Supplementary information

**Additional file 1: Supplement S1.** MTM Adaptation Framework Illustrative Case Scenario. **Table S2.** MTM Adaptability Framework Semi-structured Interview Guide.

## Data Availability

The dataset (including individual transcripts) is not publicly available due to a confidentiality agreement between the research institution and partner pharmacy chain.

## Abbreviations

CFIR: Consolidated framework for implementation research
CMR: Comprehensive medication review
CPP: Community-based pharmacist practitioners
MAP: Medication action plan
MTM: Medication therapy management
MTR: Medication therapy review
PCP: Primary care provider
PMR: Personalized medication record
PPCP: Pharmacists’ patient care process
TMR: Targeted medication review
