# Supplementary material for: Targeting adaptability to improve Medication Therapy Management (MTM) implementation in community pharmacy
Source: Implement Sci. 2019 Nov 27;14:99. doi: 10.1186/s13012-019-0946-7 (PMC6882346; doi:10.1186/s13012-019-0946-7)
Supplement: Supplementary file 1 — Additional file 1: Supplement S1. MTM Adaptation Framework Illustrative Case Scenario. Table S2. MTM Adaptability Framework Semi-structured Interview Guide. [file 13012_2019_946_MOESM1_ESM.docx]

Supplement 1: MTM Adaptation Framework Illustrative Case Scenario

A patient with uncontrolled type II diabetes mellitus (a1c=9.2%) may present for an MTM session to the pharmacist. If this patient was presenting for a CMR in a community pharmacy without access to medical records and without a pre-existing relationship with the provider, then a reasonable recommendation may be to refer that patient to either their primary care physician, and/or to a disease state management program at the same pharmacy. Alternatively, if this same patient presented to an ambulatory care pharmacist in a medical office performing CMM, a reasonable recommendation may be to modify therapy under collaborative practice agreement (CPA). However, if we modify the “inputs” to the MTM Adaptability Framework slightly, we may find the community pharmacist in the first example may have a very similar intervention to the ambulatory care pharmacist if the pharmacist providing the CMR in the community pharmacy was granted access to patient information via a health information exchange (HIE) and had an established relationship with the patient’s provider.

Supplement 2: MTM Adaptability Framework Semi-structured Interview Guide

| Domain | Question | Follow-up Questions | Probing Questions |
| --- | --- | --- | --- |
| MTM Adaptability Framework | | | |
| **Perceptions before** | Before the webinar, what were your general feelings about MTM at your pharmacy?  What was your largest barrier to MTM service delivery **prior** to the webinar? | Why did you feel this way?  How did you attempt to overcome this barrier(s)? | Anything else?  Any other thoughts? |
| **Perceptions after** | After the webinar, what were your general feelings about MTM at your pharmacy?  What part(s) of the webinar did you incorporate into your practice?  Describe how your approach to delving MTM changed after the webinar. | Why?  Why?  Were there other changes to your approach? | Any other thoughts?  Anything else to add?  Anything else? |
